# Supplementary material for: Endocytosis-mediated entry of a caterpillar effector into plants is countered by Jasmonate
Source: Nat Commun. 2023 Oct 17;14:6551. doi: 10.1038/s41467-023-42226-1 (PMC10582130; doi:10.1038/s41467-023-42226-1)
Supplement: Supplementary file 1 — Supplementary Information [file 41467_2023_42226_MOESM1_ESM.pdf]

Supporting Information for

Endocytosis-mediated entry of a caterpillar effector into plants is countered by Jasmonate

Zi-Wei Yan[1,2,7], Fang-Yan Chen[1,2,3,7], Xian Zhang[1,2], Wen-Juan Cai[4], Chun-Yu Chen[1,2], Jie Liu[1,5], Man-Ni Wu[1,2], Ning-Jing Liu[3], Bin Ma[3], Mu-Yang Wang[1], Dai-Yin Chao[3], Cai-Ji Gao[6] and Ying-Bo Mao[1,*]

[1] *CAS Key Laboratory of Insect Developmental and Evolutionary Biology, CAS Center for Excellence in Molecular Plant Sciences (CEMPS), Institute of Plant Physiology and Ecology (SIPPE), Chinese Academy of Sciences (CAS), Shanghai, China*

[2] *University of CAS, Shanghai, China*

[3] *National Key Laboratory of Plant Molecular Genetics, CEMPS/SIPPE, CAS, Shanghai, China*

[4] *Core Facility Center of CEMPS/SIPPE, CAS, Shanghai, China*

[5] *Shanghai Key Laboratory of Plant Molecular Sciences, College of Life Sciences, Shanghai Normal University, Shanghai, China*

[6] *Guangdong Provincial Key Laboratory of Biotechnology for Plant Development, School of Life Sciences, South China Normal University (SCNU), Guangzhou, China*

[7] *These authors contributed equally:* Zi-Wei Yan, Fang-Yan Chen.

**Correspondence:* Ying-Bo Mao, email: ybmao@cemps.ac.cn

Supplementary Figures

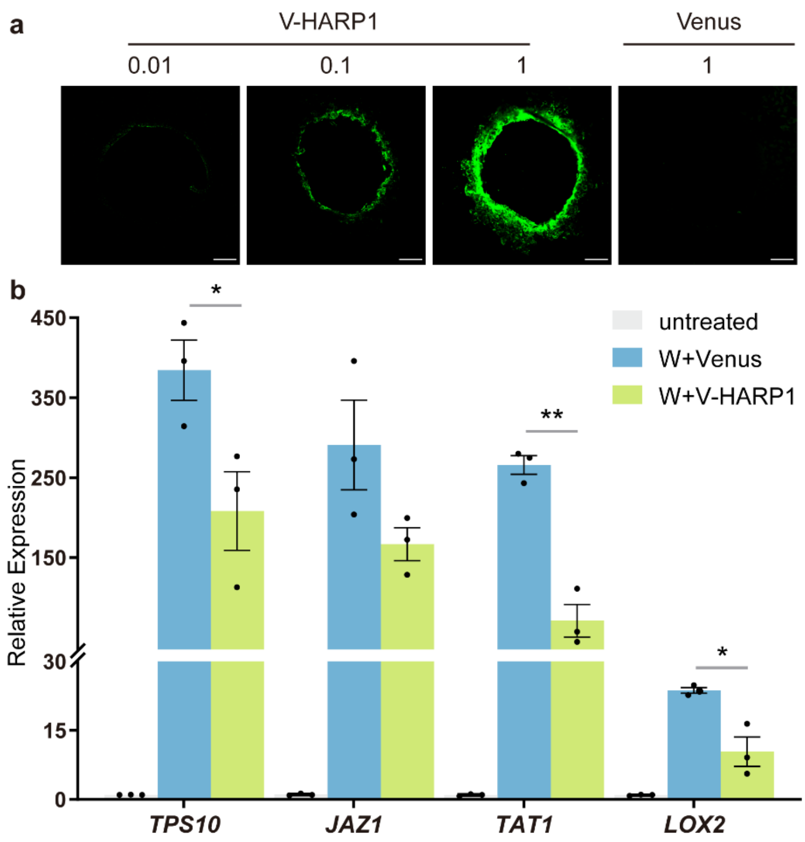

Supplementary Figure 1. Weakened plant wounding response by V-HARP1 application. (a) Confocal microscopy observation of V-HARP1 around the wounding site of *Arabidopsis* leaves. The wounded leaves were incubated with V-HARP1 (0.01, 0.1 and 1 mg/ml) and Venus (1 mg/ml) for four hours, respectively. Scale bar, 200 μm. **(b)** *Arabidopsis* leaves were wounded and painted with 1 mg/ml Venus (W+Venus, blue indicated) or V-HARP1 (W+V-HARP1, green indicated) on the wounding sites quickly. The expressions of the indicated genes were detected 4 hours post treatments by qRT-PCR. The gene expressions in the unwounded plants (untreated, gray indicated) were set to 1. Data are means ± SEM (n = 3 biological replicates) and analyzed by two-sided Student's t test (*$P < 0.05$, **$P < 0.01$). Source data are provided as a Source Data file.

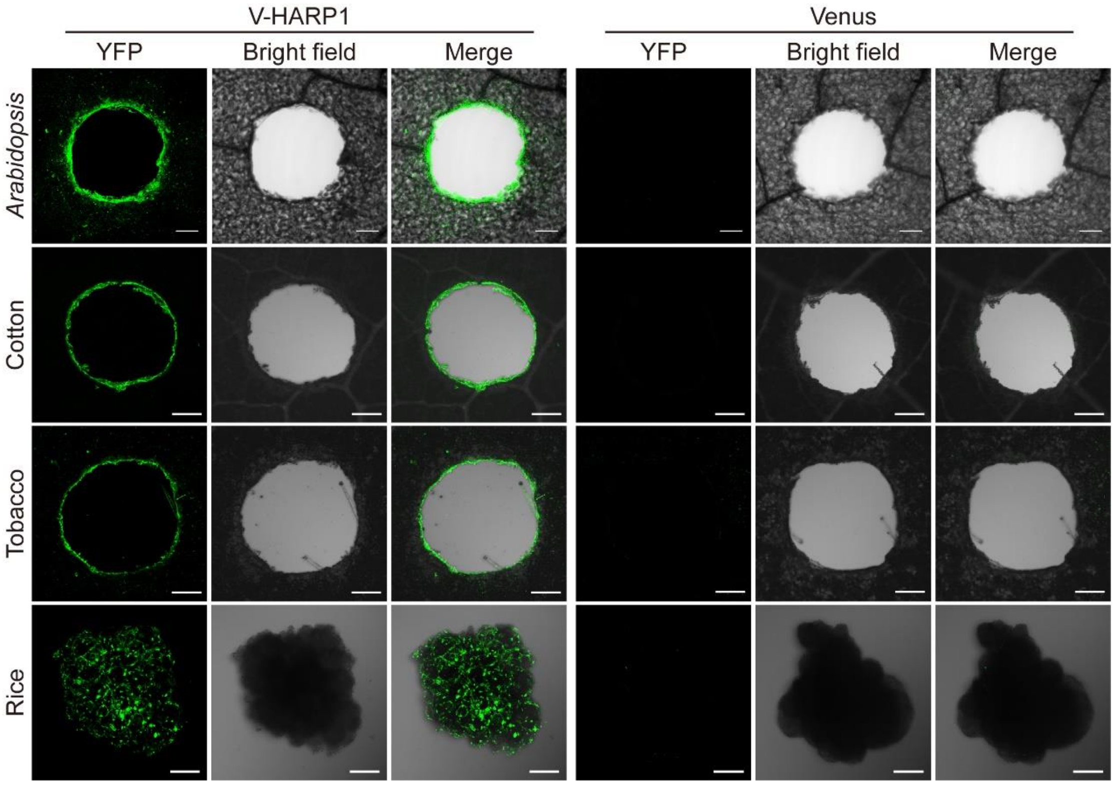

Supplementary Figure 2. Entry of V-HARP1 into *Arabidopsis*, cotton, tobacco, and rice callus. The wounded *Arabidopsis*, cotton and tobacco leaves as well as rice callus were incubated with V-HARP1 or Venus, respectively and then washed with PBS containing 0.08% BSA for 6 times. Confocal microscopy was used for detection. YFP indicates the fluorescence signal of Venus and V-HARP1. Scale bar, 200 µm.

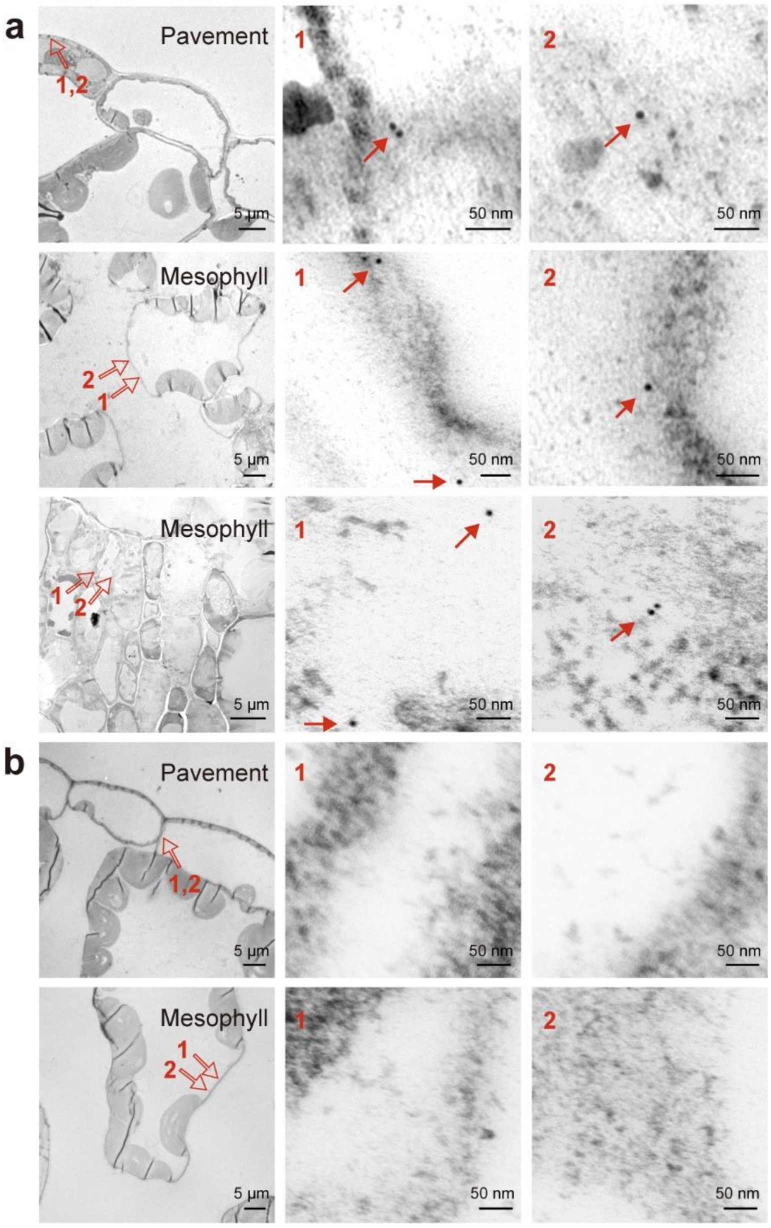

Supplementary Figure 3. It was V-HARP1 not Venus that was found in pavement and mesophyll cells under transmission electron microscope (TEM) observation. Ultrathin sections from V-HARP1 **(a)** and Venus **(b)** treated *Arabidopsis* leaves were immune-gold labeled with anti-GFP antibody. The enlarged view of the hollow arrow indicated regions (1 and 2) were shown independently. The solid arrows indicate the immune-gold labeled V-HARP1. Scale bars were indicated in each image.

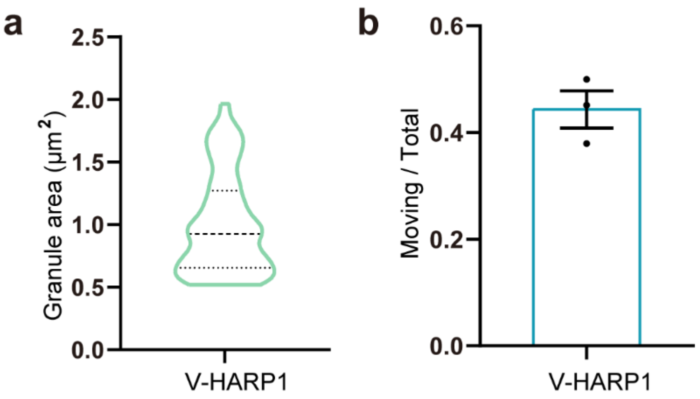

Supplementary Figure 4. Quantification of moving V-HARP1 granules. (a) Size quantification of V-HARP1 granules. The areas of about 108 granules from six independent sights were calculated in ImageJ. Data are means ± SD (n = 108). **(b)** The ratio of moving to total V-HARP1 granules. The moving V-HARP1 was tracked and counted by Olympus cellSens software. Three independent sights with total of 119 V-HARP1 granules were used for calculation. Data are means ± SEM (n = 3 biological replicates). Source data are provided as a Source Data file.

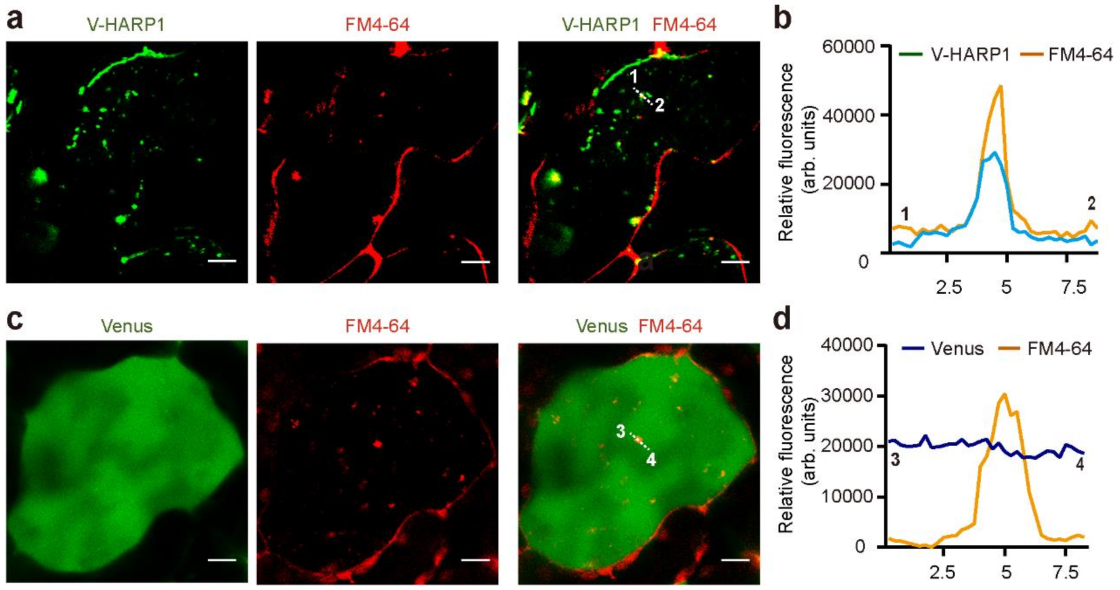

Supplementary Figure 5. V-HARP1 not Venus was observed as granules in plant leaves. *Arabidopsis* leaves were injected with V-HARP1 and Venus at the concentration of 0.2 µg/ml, respectively. And four hours post injection, the leaves were stained with FM4-64 for 30 min to mark membrane and endosomes. Scale bar, 10 µm. **(a)** Injected V-HARP1 in *Arabidopsis* leaves. **(b)** Fluorescence intensity (in arbitrary units, arb. units) of the cross sections [dotted line in **(a)**]. Blueand orange lines indicate V-HARP1 and FM4-64 fluorescence signals, respectively. **(c)** Injected Venus in *Arabidopsis* leaves. **(d)** Fluorescence intensity (in arbitrary units, arb. units) of the cross sections [dotted line in **(c)**]. Blue and orange lines indicate Venus and FM4-64 fluorescence signals, respectively.

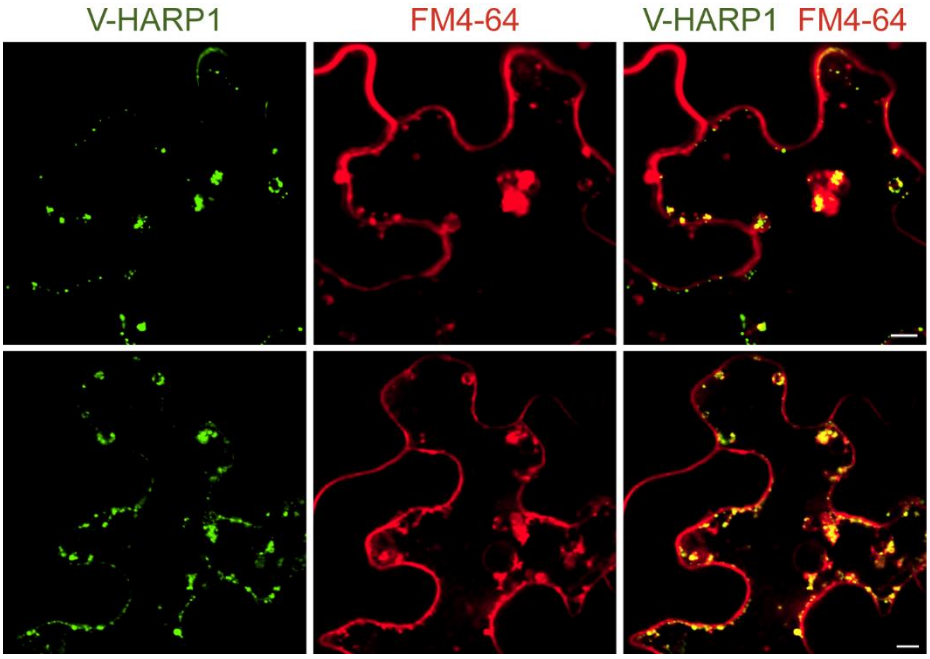

Supplementary Figure 6. V-HARP1 locates in internalized endosomes. The wounded *Arabidopsis* leaves were incubated with V-HARP1 and further stained with FM4-64 to trace internalized endosomes. Scale bar, 5 μm.

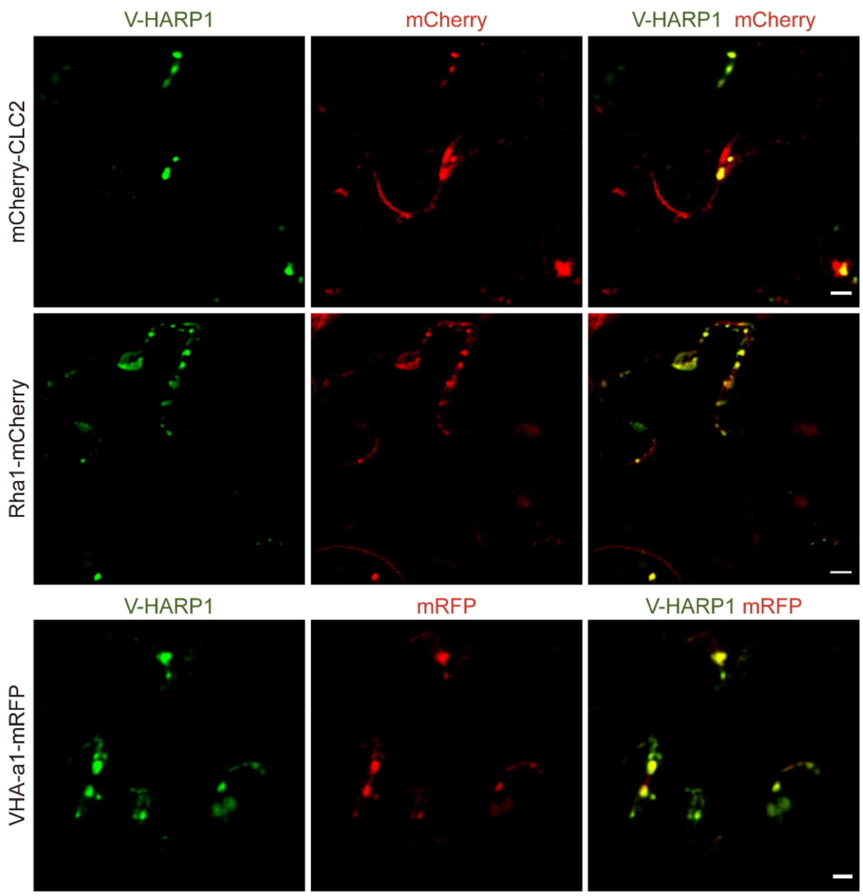

Supplementary Figure 7. V-HARP1 colocalizes with mCherry-CLC2, Rha1-mCherry, and VHA-a1-mRFP marked endosomes. The wounded leaves of indicated *Arabidopsis* plants were incubated with V-HARP1, respectively. mCherry-CLC2 and VHA-a1-mRFP indicate clathrin-coated vesicles and secretory vesicles of early endosomes, respectively. Rha1-mCherry indicates prevacuolar compartment/multivesicular body/late endosomes. Scale bar, 5 μm.

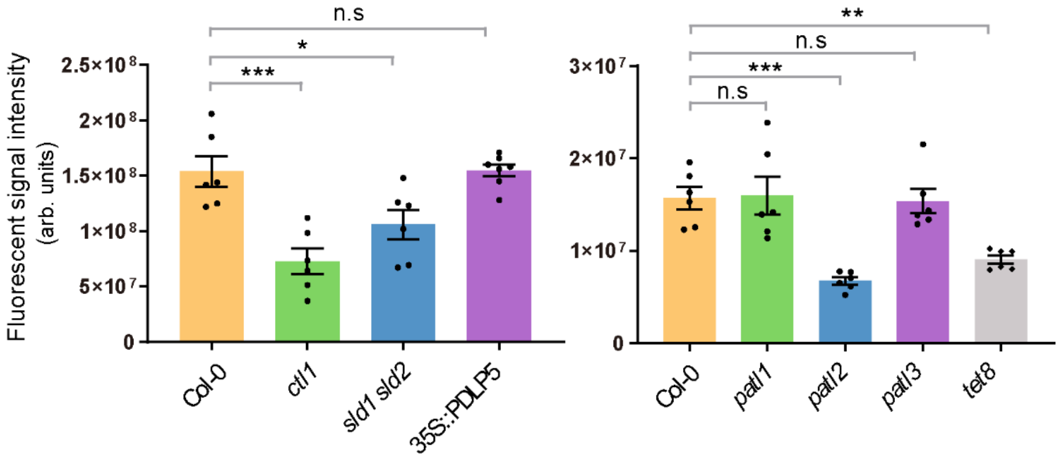

Supplementary Figure 8. Quantifications of V-HARP1 signals in Fig. 3a and 3b. The indicated *Arabidopsis* leaves were wounded and incubated with V-HARP1. 6 random sights were selected for calculation. Integrated intensity of fluorescence(in arbitrary units, arb. units) was measured in the whole sight selected. Data are means ± SEM (n = 6 biological replicates) and analyzed by one-way ANOVA with two-sided Dunnett's post hoc test (n.s: no significance, *p < 0.05, **p < 0.01, ***p < 0.001). Source data are provided as a Source Data file.

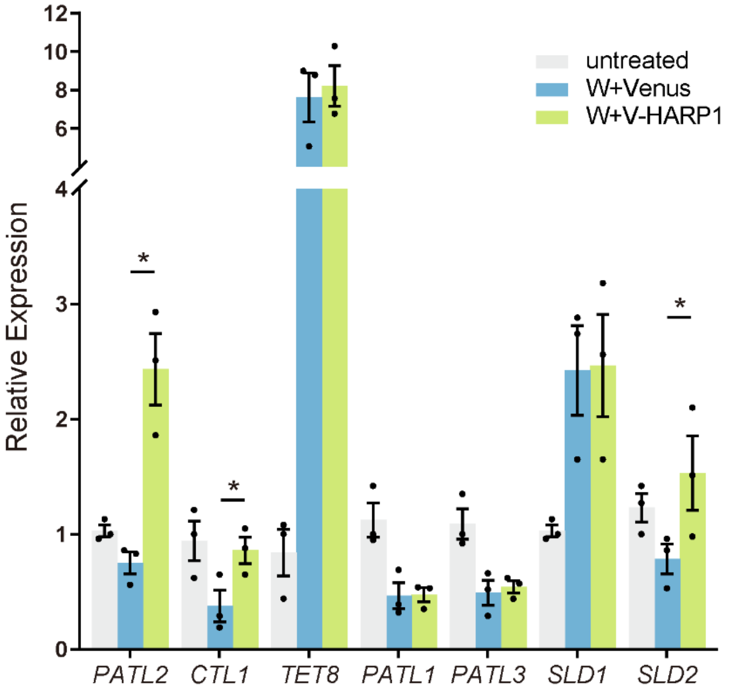

Supplementary Figure 9. V-HARP1 induces the expressions of *PATL2, CTL1* and *SLD2* in wounding response. *Arabidopsis* leaves were wounded and immediately painted with 1 mg/ml Venus (W+Venus, blue indicated) or V-HARP1 (W+V-HARP1, green indicated) on the wounding sites. Gene expressions four hours post treatments were detected by qRT-PCR. The expressions in the untreated (gray indicated) plants were set to 1. Data are means ± SEM (n = 3 biological replicates) and analyzed by two-sided Student's t test (*$P < 0.05$). Source data are provided as a Source Data file.

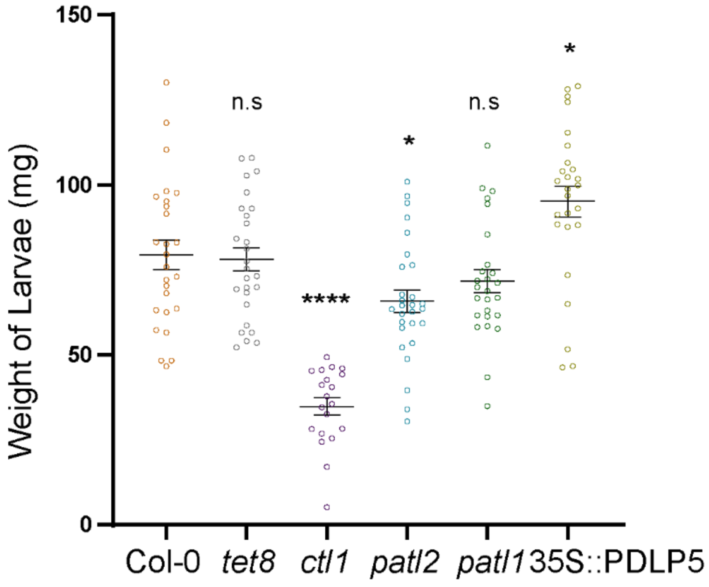

Supplementary Figure 10. The growth of *H. armigera* larvae fed on *ctl1* or *patl2* plants were inhibited. The synchronous third-instar larvae were fed on wild type (Col-0) and the indicated mutants for 4 days and subsequently weighted. Data are means ± SEM (n = 20-28) and analyzed by two-sided Student's t test (n.s: no significance, *$p < 0.05$, ****$p < 0.0001$). Source data are provided as a Source Data file.

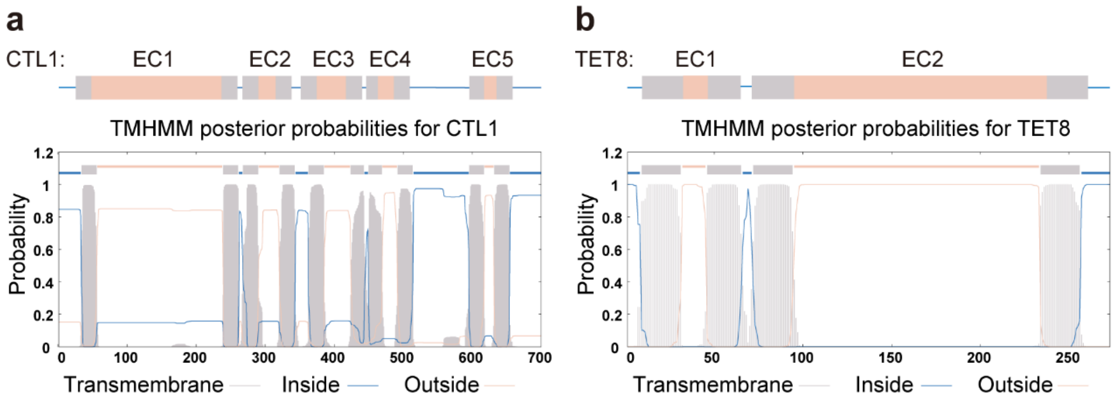

Supplementary Figure 11. Predicted extracellular loops (ECs, pink) and transmembrane helices (gray) of CTL1 and TET8. The transmembrane helices in CTL1 **(a)** and TET8 **(b)** were predicted by TMHMM (http://www.cbs.dtu.dk/services/TMHMM/).

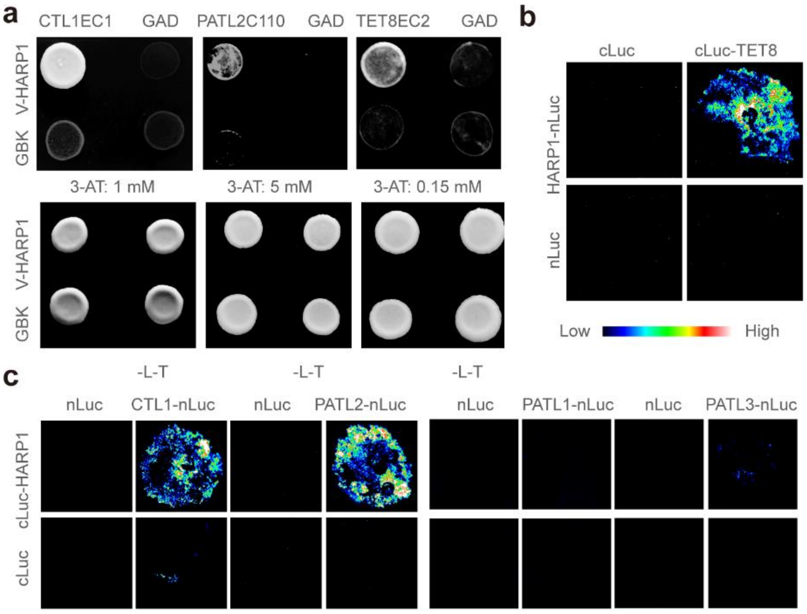

Supplementary Figure 12. HARP1 interacts with CTL1, PATL2 and TET8. (a) Yeast two-hybrid assays of CTL1EC1, PATL2C110 and TET8EC2 interaction with V-HARP1. V-HARP1 was fused to GAL4 DNA-binding domain (BD), CTL1EC1, PATL2C110 and TET8EC2 were fused to GAL4 activation domain (AD), respectively. To examine the interaction with CTL1EC1 and TET8EC2, yeast cells were grown on medium (-L-T-H) with 1 mM and 0.15 mM 3-AT, respectively. To examine the interaction with PATL2C110, yeast cells were grown on medium (-A-L-T-H) with 5 mM 3-AT. **(b-c)** Detection of HARP1 interaction with TET8 **(b)** as well as with PATLs (PATL1, 2, 3) and CTL1 **(c)** by BiLC assay. The colors ranged from white, red, green to blue indicate a strong to weak interaction. **(b)** TET8 was fused to the carboxyl-terminal half of LUCIFERASE (cLuc) and HARP1 was fused to the amino-terminal half of LUCIFERASE (nLuc). **(c)** CTL1, PATL1, PATL2 and PATL3 were fused to nLuc and HARP1 was fused to cLuc.

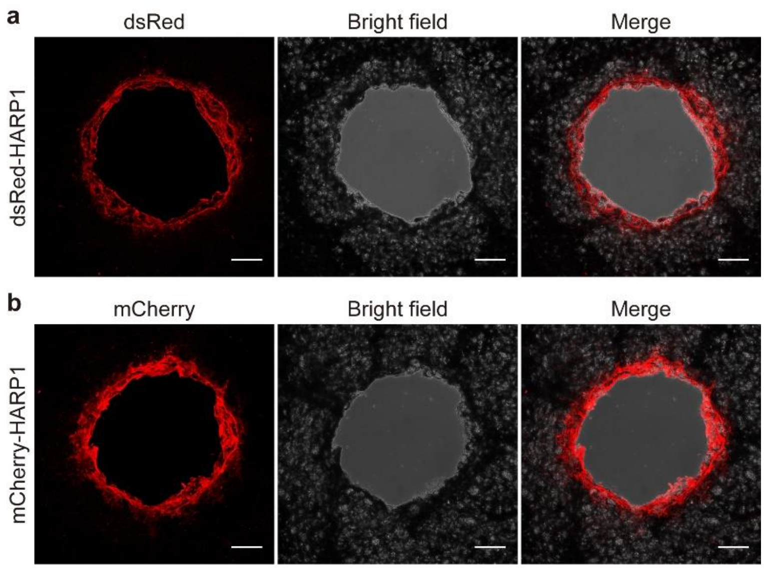

Supplementary Figure 13. Imports of dsRed-HARP1 and mCherry-HARP1 into *Arabidopsis* leaf tissues. The wounded *Arabidopsis* leaves were incubated with dsRed-HARP1 **(a)** or mCherry-HARP1 **(b)**, respectively and observed under fluorescence microscope. Scale bar, 200 μm.

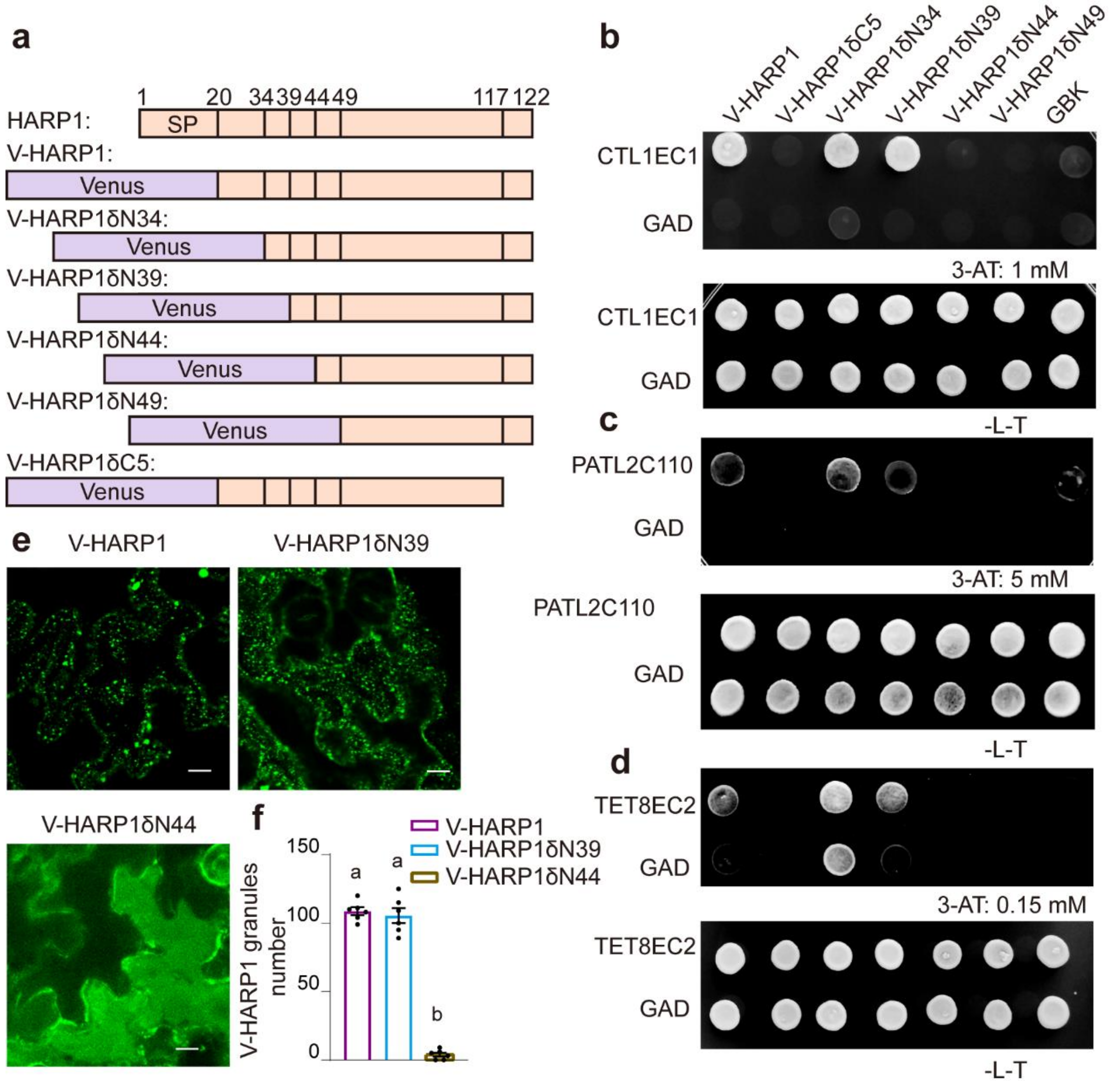

Supplementary Figure 14. The interactions of truncated V-HARP1 with CTL1EC1, PATL2C110 and TET8EC2. (a) Schematic diagram of truncated V-HARP1. **(b-d)** Yeast two-hybrid assay to examine the interactions of full length and truncated V-HARP1 with CTL1EC1 **(b)**, PATL2C110 **(c)** and TET8EC2 **(d)**. V-HARP1 and its indicated truncated forms were fused to GAL4 DNA-binding domain (BD), CTL1EC1, PATL2C110 and TET8EC2 were fused to GAL4 activation domain (AD), respectively. Yeast cells were examined as described in Supplementary Figure 12. (**e**) Confocal image of V-HARP1, V-HARP1δN39 and HARP1δN44 which were delivered into Arabidopsis leaves by injection. Scale bar, 5 μm. **(f)** Quantification of the V-HARP1 granules number in (**e**). 6 random sights were selected to count the

granule numbers of V-HARP1 (purple indicated), V-HARP1δN39 (blue indicated) and HARP1δN44 (brown indicated). Data are means ± SEM (n =6 biological replicates) and analyzed by one-way ANOVA with two-sided Dunnett's post hoc test. Different letters indicate significant differences ($P < 0.05$). Source data are provided as a Source Data file.

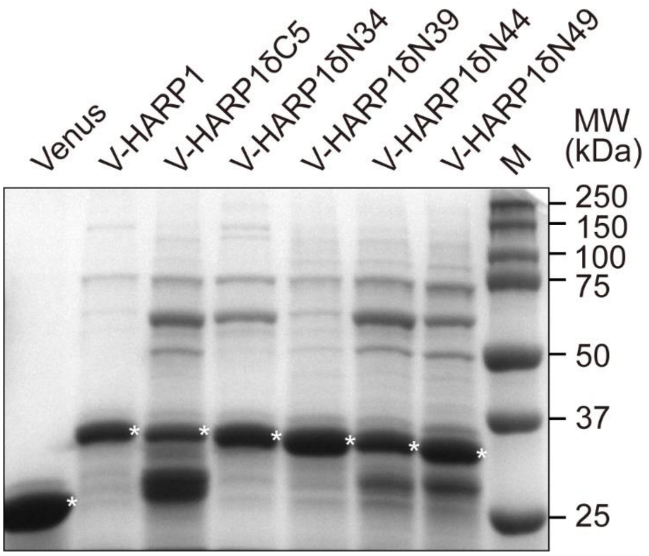

Supplementary Figure 15. Coomassie blue staining of Venus and indicated truncated V-HARP1s mentioned in Fig. 4f. The procaryotically expressed proteins were purified and subjected to SDS agarose gel electrophoresis. * Stands for the target band of the indicated protein.

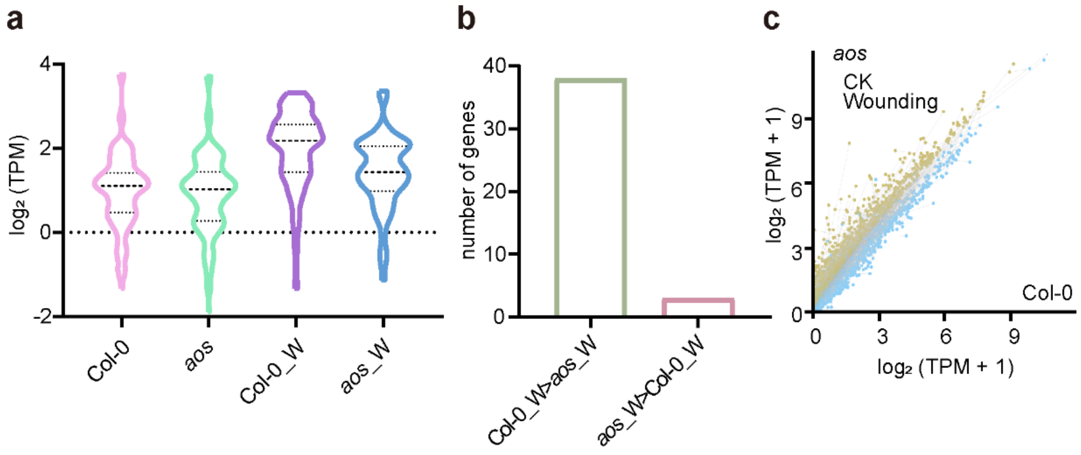

Supplementary Figure 16. Transcriptomic analysis of gene expressions in Col-0 and *aos*. (a) Expression of 92 genes annotated as response to JA which were detected in our transcriptome data (Supplementary Data file2). CK stands for untreated plants and W stands for plants two hours post wounding. **(b)** The expressions of most of genes as described in **(a)** were higher in Col-0 than in *aos* after wounding. **(c)** Scatter plot analysis of the 1121 genes with higher expression in *aos* than in Col-0 after wounding (*aos*_W / Col-0_W > 2, FDR < 0.05).

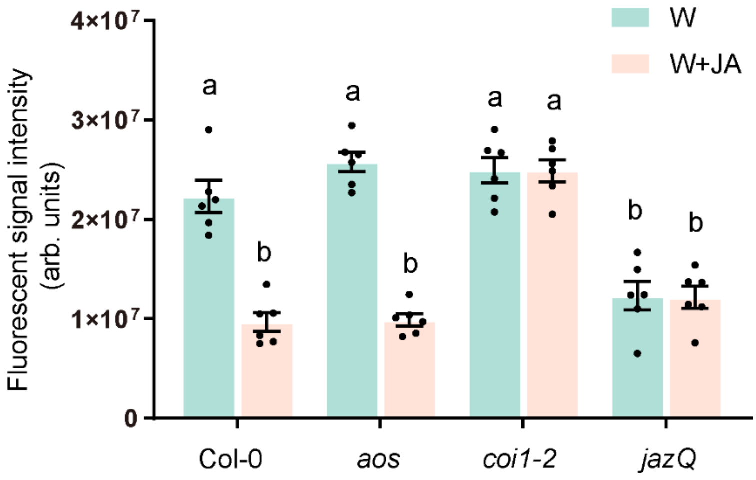

Supplementary Figure 17. Quantification of V-HARP1 signals in Fig. 6a. The fluorescent signal intensity (in arbitrary units, arb. units) of V-HARP1 in leaves of Col-0, *aos*, *coi1-2* and *jazQ* were measured. Light blue and light pink box indicate Ethanol (W) or 50 µM MeJA (W+JA) treatment, respectively.Data are means ± SEM (n = 6 biological replicates) and analyzed by two-way ANOVA followed by multiple comparisons with Fisher's LSD test. Different letters indicate significant differences ($P < 0.05$). Source data are provided as a Source Data file.

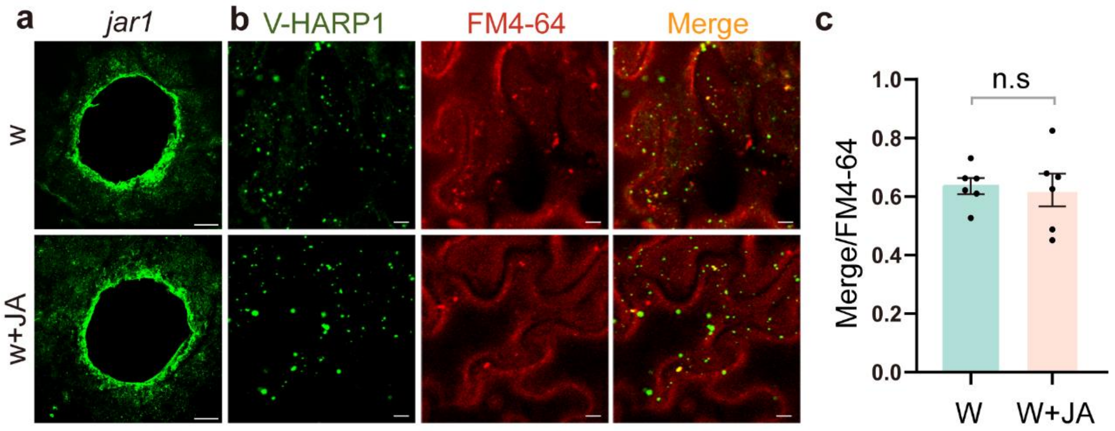

Supplementary Figure 18. **JA has no detectible impacts on HARP1 import into jar1.** (**a**) Confocal image of the V-HARP1 signals around the wounding sites of the *jar1* leaves. Leaves of *jar1* were pretreated with Ethanol (W) or 50 μM MeJA (W+JA) for two hours and subsequently incubated with V-HARP1. Scale bar: 200 μm. (**b**) V-HARP1-loaded endosomes in the plants. Plants were treated as described in (**a**). The internalized endosomes were traced by FM4-64. Scale bar: 5 μm. (**c**) Quantification of endosomes from (**b**). 6 random sights were selected to count the number of endosomes. Data are means ± SEM (*n* = 6 biological replicates) and analyzed by two-way ANOVA followed by multiple comparisons with Fisher's LSD test (n.s: no significance). Source data are provided as a Source Data file.

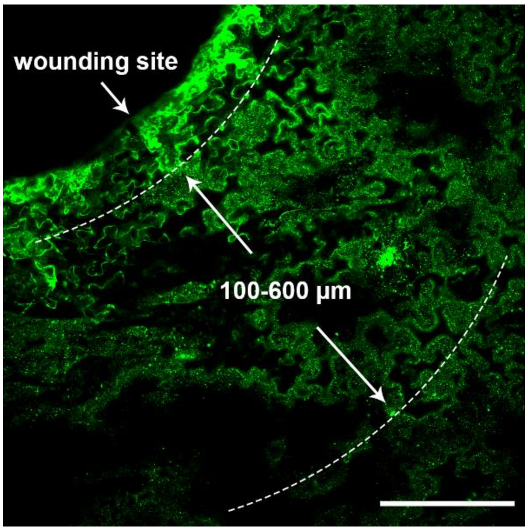

Supplementary Figure 19. Regions used for observing the subcellular localization and movement of HARP1. The wounded *Arabidopsis* leaves were incubated with V-HARP1 for four hours. Samples were washed with PBS containing 0.08% BSA for at least 5 times (20 minutes for each time) and then were detected under confocal microscopy. The dotted lines indicated the area where the subcellular localization and movement of HARP1 were observed. Scale bar, 200 μm.

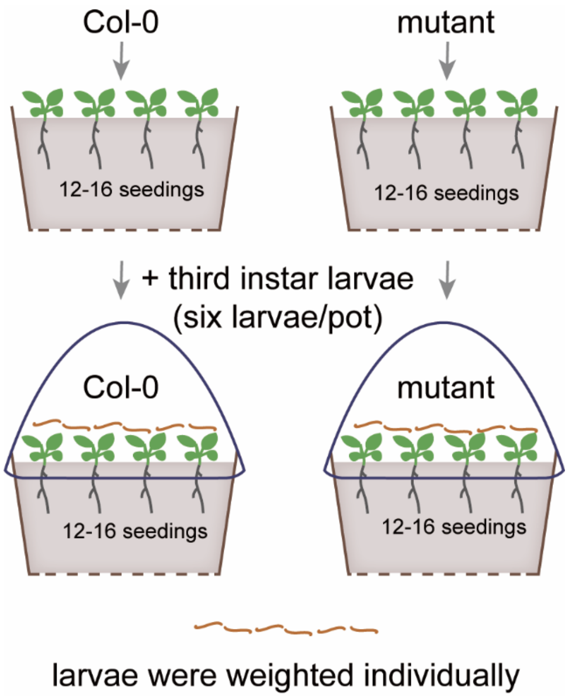

Supplementary Figure 20. Diagram of cotton bollworm feeding test. About 12-16 seedlings of *Arabidopsis* were planted in one pot. 5-6 pots of three-week-old plants of wild type (Col-0), *tet8*, *ctl1*, and *patl2* were used for insect feeding test. About 5-6 3rd instar larvae were put into one pot and covered with a plastic cover. After feeding for 3-4 days, larvae were weighted individually.
