# Supplementary material for: Endocytosis-mediated entry of a caterpillar effector into plants is countered by Jasmonate
Source: Nat Commun. 2023 Oct 17;14:6551. doi: 10.1038/s41467-023-42226-1 (PMC10582130; doi:10.1038/s41467-023-42226-1)
Supplement: Supplementary file 3 — Reporting Summary [file 41467_2023_42226_MOESM3_ESM.pdf]

nature portfolio

Corresponding author(s): Ying-Bo Mao

Last updated by author(s): 2023/09/28

Reporting Summary

Nature Portfolio wishes to improve the reproducibility of the work that we publish. This form provides structure for consistency and transparency in reporting. For further information on Nature Portfolio policies, see our Editorial Policies and the Editorial Policy Checklist.

Statistics

For all statistical analyses, confirm that the following items are present in the figure legend, table legend, main text, or Methods section.

n/a	Confirmed	
☐	☒	The exact sample size (*n*) for each experimental group/condition, given as a discrete number and unit of measurement
☐	☒	A statement on whether measurements were taken from distinct samples or whether the same sample was measured repeatedly
☐	☒	The statistical test(s) used AND whether they are one- or two-sided<br>*Only common tests should be described solely by name; describe more complex techniques in the Methods section.*
☒	☐	A description of all covariates tested
☐	☒	A description of any assumptions or corrections, such as tests of normality and adjustment for multiple comparisons
☐	☒	A full description of the statistical parameters including central tendency (e.g. means) or other basic estimates (e.g. regression coefficient) AND variation (e.g. standard deviation) or associated estimates of uncertainty (e.g. confidence intervals)
☐	☒	For null hypothesis testing, the test statistic (e.g. *F*, *t*, *r*) with confidence intervals, effect sizes, degrees of freedom and *P* value noted<br>*Give P values as exact values whenever suitable.*
☒	☐	For Bayesian analysis, information on the choice of priors and Markov chain Monte Carlo settings
☒	☐	For hierarchical and complex designs, identification of the appropriate level for tests and full reporting of outcomes
☒	☐	Estimates of effect sizes (e.g. Cohen's *d*, Pearson's *r*), indicating how they were calculated

Our web collection on statistics for biologists contains articles on many of the points above.

Software and code

Policy information about availability of computer code

Data collection	No software was used for data collection.
Data analysis	No software was used

For manuscripts utilizing custom algorithms or software that are central to the research but not yet described in published literature, software must be made available to editors and reviewers. We strongly encourage code deposition in a community repository (e.g. GitHub). See the Nature Portfolio guidelines for submitting code & software for further information.

Data

Policy information about availability of data

All manuscripts must include a data availability statement. This statement should provide the following information, where applicable:

- Accession codes, unique identifiers, or web links for publicly available datasets
- A description of any restrictions on data availability
- For clinical datasets or third party data, please ensure that the statement adheres to our policy

The RNA-seq data that support the findings of this study have been deposited in the National Center for Biotechnology Information (NCBI) under the following accession codes: PRJNA760932, https://www.ncbi.nlm.nih.gov/sra/?term=PRJNA760932. The relevant raw data from each figure are provided in the Source Data file. All data supporting the findings of this study are included in this article, Supplementary Information and Source Data file. Source Data are provided with this paper.

Research involving human participants, their data, or biological material

Policy information about studies with human participants or human data. See also policy information about sex, gender (identity/presentation), and sexual orientation and race, ethnicity and racism.

Reporting on sex and gender	N/A
Reporting on race, ethnicity, or other socially relevant groupings	N/A
Population characteristics	N/A
Recruitment	N/A
Ethics oversight	N/A

Note that full information on the approval of the study protocol must also be provided in the manuscript.

Field-specific reporting

Please select the one below that is the best fit for your research. If you are not sure, read the appropriate sections before making your selection.

- [x] Life sciences
- [ ] Behavioural & social sciences
- [ ] Ecological, evolutionary & environmental sciences

For a reference copy of the document with all sections, see nature.com/documents/nr-reporting-summary-flat.pdf

Life sciences study design

All studies must disclose on these points even when the disclosure is negative.

Sample size	For confocal microscopy, the scale bar was given in each figure.<br>For RNA-seq and qRT-PCR experiments, at least three biological triplicates with technical triplicates were performed.
Data exclusions	No data were excluded.
Replication	In Fig. 1c-d, six random sights from 3-5 plants were selected for calculation.<br>In Fig. 2b, seven random sights from leaves of 4-5 plants were selected for calculation.<br>In Fig. 3d, six biological replicates were selected for calculation.<br>In Fig. 4e, six random sights from 3-5 plants were selected for calculation.<br>In Fig. 6c-e, six biological replicates were selected to count the number of endosomes.<br>In Fig. S4a, three independent sights with total of 108 V-HARP1 granules were used for calculation.<br>In Fig. S4b, three biological replicates were selected for calculation.<br>In Fig. S8, six biological replicates were selected for calculation.<br>In Fig. 14f, six biological replicates were selected for calculation.<br>In Fig. S17, six biological replicates were selected for calculation.<br>In Fig. 18c, six biological replicates were selected for calculation.<br>For insect feeding test (Fig. S10), about 30 synchronous third instar larvae were fed on 60 Arabidopsis plants of 20 days old.<br>For RNA-seq experiments, three biological replicates were performed.<br>For qRT-PCR assay, at least three technical replicates were performed.
Randomization	The samples were randomly allocated into experimental groups.
Blinding	Investigators were blinded to group allocation.

Reporting for specific materials, systems and methods

We require information from authors about some types of materials, experimental systems and methods used in many studies. Here, indicate whether each material, system or method listed is relevant to your study. If you are not sure if a list item applies to your research, read the appropriate section before selecting a response.

Materials & experimental systems

n/a	Involved in the study
☐	☒ Antibodies
☒	☐ Eukaryotic cell lines
☒	☐ Palaeontology and archaeology
☐	☒ Animals and other organisms
☒	☐ Clinical data
☒	☐ Dual use research of concern
☐	☒ Plants

Methods

n/a	Involved in the study
☒	☐ ChIP-seq
☒	☐ Flow cytometry
☒	☐ MRI-based neuroimaging

Antibodies

Antibodies used	Antibody used in pull-down assay: Anti-His antibody (ABclonal; dilution, 1:2,500); Anti-GST antibody (ABclonal; dilution, 1:2,500); Anti-Flag antibody (ABclonal; dilution, 1:2,500)<br>Antibody used in Immuno-localization: Anti-GFP antibody (ZSGB-BIO, 1:50 diluted)<br>Antibody used in whole amount immunohistochemistry: Anti-HARP1 antibody36 (1:200 dilution). HARP1 antibody were generated by Prof. Xia's lab in Xiamen University, China(Chen et al., PNAS, 2019)<br>Antibody used in immunobolt assay: Anti-HARP1 antibody (1:1000 dilution)
Validation	Mouse monoclonal Anti-His (ABclonal, #AE003), Anti-GST antibody(ABclonal, #AE001), Anti-Flag antibody(ABclonal, #AE005), and Anti-GST antibody (ZSGB-BIO, #2955) were purchased and validated by the company. Mouse monoclonal Anti-HARP1 antibody were validated by Chen et al., PNAS, 2019.

Animals and other research organisms

Policy information about studies involving animals; ARRIVE guidelines recommended for reporting animal research, and Sex and Gender in Research

Laboratory animals	The cotton bollworm (Helicoverpa armigera) from 2nd to 4th instar larvae were used.
Wild animals	No wild animals were used in the study.
Reporting on sex	No sex-based analyze was performed.
Field-collected samples	No field collected samples were used in the study.
Ethics oversight	No ethical approval or guidance was required.

Note that full information on the approval of the study protocol must also be provided in the manuscript.
