# Supplementary material for: Endocytosis-mediated entry of a caterpillar effector into plants is countered by Jasmonate
Source: Nat Commun. 2023 Oct 17;14:6551. doi: 10.1038/s41467-023-42226-1 (PMC10582130; doi:10.1038/s41467-023-42226-1)
Supplement: Supplementary file 4 — Description of Additional Supplementary Files [file 41467_2023_42226_MOESM4_ESM.pdf]

Description of Additional Supplementary Files

Supplementary Data 1. Primers used in this investigation.

Supplementary Data 2. RNA-seq analysis of wild type (Col-0) and aos without and with wounding treatment.

Supplementary Movie 1. V-HARP1 is moving. The wounded Arabidopsis leaves were incubated with V-HARP1. Time-lapse sequences were captured to trace the movement of V-HARP1. And the trails of the selected moving granules were marked. Scale bar, 20 μm. Time is shown at the bottom left.

Supplementary Movie 2-3. Movements of V-HARP1-loaded endosomes in plant. The wounded Arabidopsis leaves were incubated with V-HARP1. Time-lapse sequences were captured to trace the movements of V-HARP1 in plant cells. FM4-64 (Red) was used to trace internalized endosomes. Scale bar, 5 μm. Time is shown at the bottom left.
